# Supplementary material for: Caregiver-Rated Inappropriate Speech and Post-cTBS Motor Cortical Facilitation in Autism: A Pilot Biomarker Study
Source: medRxiv. 2026 Sep 12:2026.09.10.26362749. Preprint. [Version 1] doi: 10.64898/2026.09.10.26362749 (PMC13622877; doi:10.64898/2026.09.10.26362749)
Supplement: Supplementary file 1 [file media-7.pdf]

Supplementary Figure 1: Participant flow through enrollment, diagnostic TMS procedures, and analytic cohorts

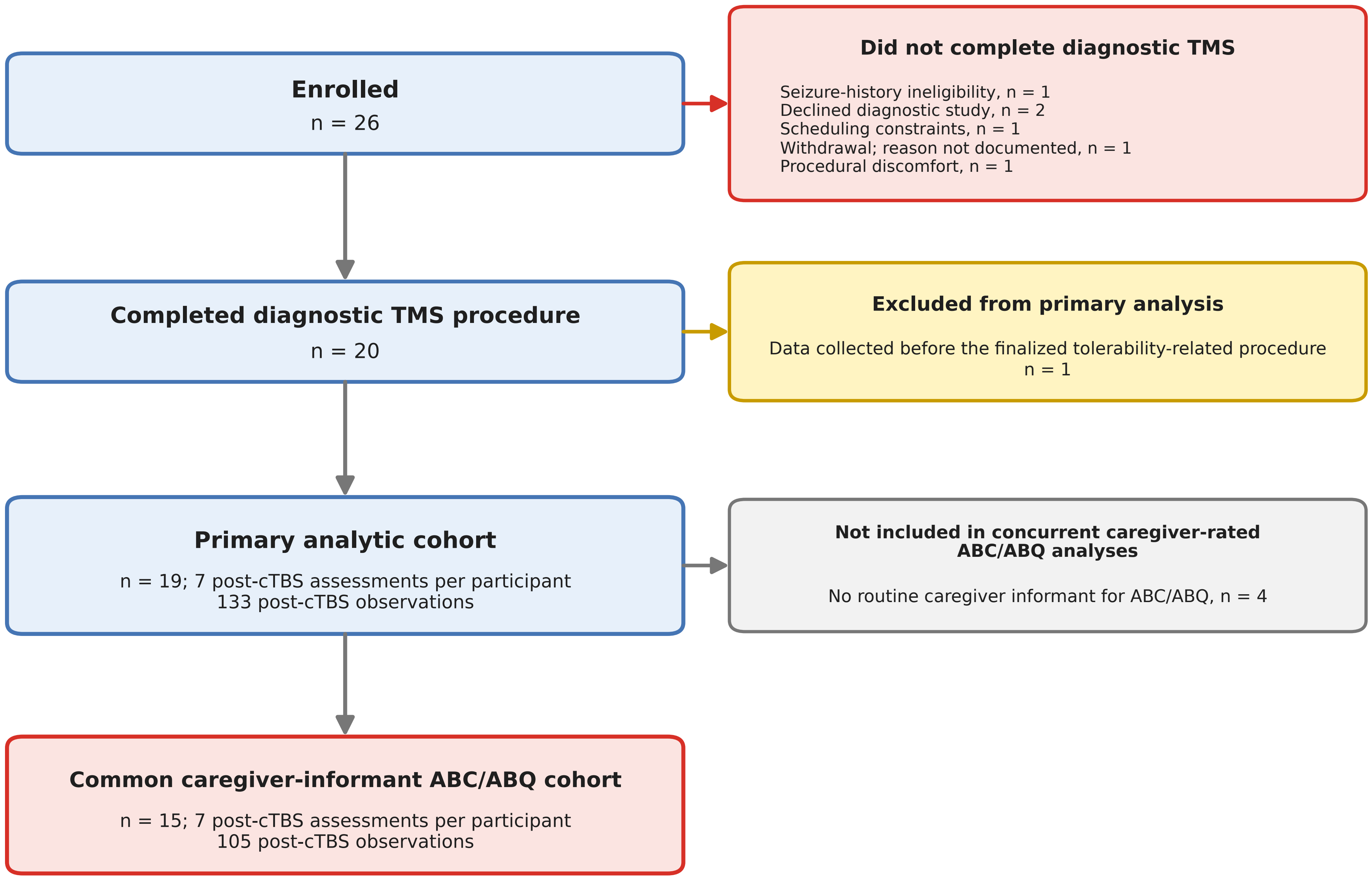

No randomization, treatment allocation, or concurrent comparator was used. All participants completing the finalized procedure followed the same diagnostic TMS protocol.

ABC, Aberrant Behavior Checklist; ABQ, Attenuated Behavior Questionnaire; cTBS, continuous theta-burst stimulation; TMS, transcranial magnetic stimulation.
